# Insular activation during reward anticipation reflects duration of illness in abstinent pathological gamblers

**DOI:** 10.3389/fpsyg.2014.01013

**Published:** 2014-09-09

**Authors:** Kosuke Tsurumi, Ryosaku Kawada, Naoto Yokoyama, Genichi Sugihara, Nobukatsu Sawamoto, Toshihiko Aso, Hidenao Fukuyama, Toshiya Murai, Hidehiko Takahashi

**Affiliations:** ^1^Department of Psychiatry, Kyoto University Graduate School of MedicineKyoto, Japan; ^2^Department of Neurology, Kyoto University Graduate School of MedicineKyoto, Japan; ^3^Human Brain Research Center, Kyoto University Graduate School of MedicineKyoto, Japan

**Keywords:** pathological gambling, addiction, insula, neuroimaging, fMRI, reward

## Abstract

Pathological gambling (PG) is a chronic mental disorder characterized by a difficulty restraining gambling behavior despite negative consequences. Although brain abnormalities in patients with substance use disorders are caused by repetitive drug use and recover partly with drug abstinence, the relationship between brain activity and duration of illness or abstinence of gambling behavior in PG patients remains unclear. Here, using functional magnetic resonance imaging, we compared the brain activity of 23 PG patients recruited from a treatment facility with 27 demographically-matched healthy control subjects during reward anticipation, and examined the correlations between brain activity and duration of illness or abstinence in PG patients. During reward anticipation, PG patients showed decreased activity compared to healthy controls in a broad range of the reward system regions, including the insula cortex. In PG patients, activation in the left insula showed a significant negative correlation with illness duration. Our findings suggest that insular activation during reward anticipation may serve as a marker of progression of pathological gambling.

## Introduction

Pathological gambling (PG) is a chronic mental disorder, in which an individual cannot stop gambling behavior in spite of severe negative consequences such as huge debt, job loss, and family break-up. PG is more prevalent in Japan (5.5%), especially in males (9.6%), than in Western countries (1–3%) (http://mhlw-grants.niph.go.jp/niph/search/NIDD00.do?resrchNum=200926008B#selectGaiyou). Although PG is a relatively common disorder, it was still being classified under “Impulse-Control Disorders Not Elsewhere Classified” in DSM-IV-TR and received less attention than substance use disorders (American Psychiatric Association, 2000). However, accumulating evidence suggests that PG, a behavioral addiction, has many similarities (e.g., risky decision making and neural underpinnings) with substance use disorders (Bechara, 2003; Potenza, 2008). This has prompted the classification of PG within “Substance-Related and Addictive Disorders” along with substance use disorders in DSM-5 (American Psychiatric Association, 2013).

Brain imaging studies have suggested that the pathophysiology of addiction is linked to abnormalities in the reward circuit, particularly in the striatum (Everitt et al., 2008) and midbrain (Morgane et al., 2005). The close relationship between striatum/midbrain and addiction has been examined from various points of view, including cue reactivity (Vezina, 2004), craving (Paulus, 2007), memory (Robbins and Everitt, 2002), and so forth. In addition, the insula, which has dense reciprocal connections with the striatum (Haber and Knutson, 2010), also seems to play an important role in addiction (Naqvi et al., 2007; Goldstein et al., 2009), including PG (Clark et al., 2014). This notion is supported by studies that showed a relationship between the insula and risk behavior (Damasio, 1994; Kuhnen and Knutson, 2005; Xue et al., 2010). Insular activation has been related to monetary loss (Pessiglione et al., 2006; Liu et al., 2007), and this region was also shown to be activated along with the striatum during gain anticipation in humans (Samanez-Larkin et al., 2007; Liu et al., 2011) and in rats (Kesner and Gilbert, 2007). During monetary reward anticipation, PG patients showed reduced activation in the striatum compared to healthy subjects (Balodis et al., 2012; Choi et al., 2012). On the other hand, decreased insular activity predicted relapse in addicted populations during different kinds of cognitive tasks (Paulus et al., 2005; Seo et al., 2013).

Previous functional neuroimaging studies have targeted altered brain activation in current PG patients. Thus, it is unclear whether the abnormalities in PG patients are transient characteristics resulting from excessive gambling behavior or continue even after abstinence. Studies of substance use disorders (such as cocaine and nicotine abuse) have reported that structural abnormalities (i.e., reduced gray and white matter volumes) (Bjork et al., 2003; Ersche et al., 2011) and decreased brain activation (Chang et al., 2006; Rose et al., 2012) were more pronounced in patients with longer illness duration. On the other hand, partial recovery of brain volume reduction has been reported in abstinent patients of substance use disorders (Makris et al., 2008; Xu et al., 2010). Improvements in brain function have also been indicated in abstinent methamphetamine- and cocaine-dependent individuals (Volkow et al., 2001; Brewer et al., 2008), and in abstinent cocaine-dependent rats (Hollander and Carelli, 2005). Although studies of abstinent substance users are useful for the investigation of addictive behavior in general, there remains a question as to whether recovery of brain abnormalities is due to withdrawal from repeated exposure to toxic substances (e.g., substance-specific neuronal damage) or to discontinuation of addictive behavior *per se* (e.g., dopamine-mediated neuroadaptation) (Nestler, 2005). Studying brain abnormalities in abstinent behavioral addiction including PG enables us to exclude possible effects of exposure to toxic substances, which should provide important insight that can lead to a better understanding of addiction. Although a detailed mechanism of behavioral addiction is less studied compared to drug addiction, recent animal and human studies suggested that dopamine-mediated neuroadaptation is induced by gambling addiction as well (Boileau et al., 2013; Zack et al., 2014). Thus, it is plausible that gambling (abstinence) history has effects on brain alteration, similar to drug addiction.

We hypothesized that (a) PG patients would show blunted striatal, insular and midbrain activation compared with healthy control subjects during a non-specific (i.e., unrelated to gambling) reward anticipation task; (b) activation in the striatum, insula and midbrain would show a negative correlation with the duration of illness; and (c) striatal, insular and midbrain activation would show a positive correlation with the duration of abstinence.

## Materials and methods

### Subjects

The PG group comprised 24 male patients who were referred to a treatment facility (Serenity Park Japan). The healthy control (HC) group consisted of 27 healthy male individuals matched to the PG group with respect to age. All of the subjects were recruited from the local community via word of mouth. One PG patient was excluded from the analyses because his duration of abstinence (36 months) was much longer than the other PG patients (range = 2.5–12 months, mean = 6.5, *SD* = 3.84). Finally, 23 PG patients (22 right-handed) and 27 HC subjects (26 right-handed) were analyzed. All patients met the criteria for PG according to the DSM-IV-TR, and PG symptoms were investigated with the Structured Clinical Interview for Pathological Gambling (SCI-PG) (Grant et al., 2004). Comorbid disorders were screened with the Structured Clinical Interview for DSM-IV. Three PG patients had depression, and one had attention deficit/hyperactivity disorder. All patients were drug-free for at least three months before scanning and were physically healthy at the time of the assessments. None of the patients had a history of neurological injury or disease, severe medical diseases, or illegal substance abuse that may affect brain function. Serenity Park Japan is a residential facility where patients receive 12-step-based psychological therapy. All patients were medication-free and were scanned after they had completed at least one cycle of 12-step-based intervention (about 1 month). HC subjects were also evaluated with the Structured Clinical Interview for DSM-IV, and were found to have no history of any psychiatric disorders. They had no history of neurological injury or disease, severe medical diseases, or substance abuse that may affect brain function. Demographic data of all subjects were collected with respect to age, handedness, education level, smoking status, and gambling severity. Smoking status and gambling severity were evaluated with the Fagerström Test for Nicotine Dependence (FTND) and the South Oaks Gambling Screen (SOGS), respectively. Duration of illness and abstinence were determined by questioning of the PG patients. This study was approved by the Committee on Medical Ethics of Kyoto University and was carried out in accordance with the Code of Ethics of the World Medical Association. After offering a complete description of the study, written informed consent was obtained from all subjects.

### Paradigm

The subjects completed a modified version of the monetary incentive delay task (MIDT), referring to the modification by Marutani et al. (2011), based on the original version developed by Knutson et al. (2000). Because monetary reward could be perceived as an addiction-related cue for PG patients, which might confound results (Limbrick-Oldfield et al., 2013), we used points instead of money to examine brain activation during non-specific (i.e., non-gamble-related) reward anticipation. Subjects finished a short version (3 min) of the task in order to understand the procedure before entering the scanner. MIDT consisted of 90 trials (5–6 s per trial), with inter-trial intervals of 2 s. During each trial, subjects saw one of three cues for 500 ms, followed by crosshairs for 2500–3500 ms, and then responded to a square target with a button press. If the subjects pressed a button within a variable length of time (100 or 500 ms), they would see the word “success,” otherwise they would see “failure.” A symbol indicated the amount of points accumulated if the subject succeeded in the trials. There were three types of symbols: circle, circle with one bar, and circle with three bars, indicating 0, 100, and 500 points, respectively.

### fMRI acquisition

All subjects underwent MRI scans with a 3-T whole-body scanner equipped with an 8-channel phased array head coil (Trio, Siemens, Erlangen, Germany). First, field maps with a double-echo fast low-angle shot (FLASH) sequence were acquired. The image-acquisition parameters were as follows: repetition time (TR) = 511 ms; echo time (TE) = 5.19 and 7.65 ms; flip angle (FA) = 60°; field of view (FOV) = 192 × 192 mm; matrix = 64 × 64; 38 interleaved axial slices with 3-mm thickness without gaps (3-mm cubic voxels). Functional images were obtained in a T2^*^-weighted gradient-echo -planar imaging sequence with prospective motion correction. The image-acquisition parameters were as follows: *TR* = 2400 ms; *TE* = 30 ms; *FA* = 90°; FOV = 192 × 192 mm; matrix = 64 × 64; 38 interleaved axial slices with 3-mm thickness without gaps (3-mm cubic voxels). The first two volumes were discarded to allow for signal stabilization. Each run included 283 volumes. Stimulus presentation and response collection were performed using the E-Prime 2.0 Professional software (Psychology Software Tools, Sharpsburg, PA, USA). Each subject lay supine on a scanner bed, with a button-response device held in the right hand. The subjects viewed visual stimuli that were back-projected onto a LED display through a built-in mirror. Foam pads were used to minimize head motion.

### fMRI analysis

The fMRI data were analyzed with SPM8 (Wellcome Department of Imaging Neuroscience, University of London, London, UK) on MATLAB 2010b (MathWorks, Natick, MA, USA). The functional images were corrected for differences in slice-acquisition timing. Timing-corrected images were spatially realigned to the first image of the initial run to adjust for residual head movements, and were unwarped to correct for static distortions using the Fieldmap toolbox (Hutton et al., 2002) in SPM8. The realigned and unwarped images were spatially normalized to fit to the EPI template provided in SPM8. Subsequently, all images were smoothed with an isotropic Gaussian kernel of 8 mm full-width at half-maximum. Each of the three cue and two outcome conditions was separately modeled as regressors for first-level multi-regression analysis. Motion parameters were included as additional regressors of no interest. This analysis was performed for each subject to test the correlation between the MRI signal and a train of delta functions (representing event onsets) convolved with the canonical hemodynamic response function. Low frequency noise was removed using a high-pass filter with a cutoff of 128 s. By applying the appropriate linear contrast to the parameter estimates, mean-effect images reflecting the magnitude of correlation between the signals and the model of interest were computed for every 5 conditions (i.e., 0, 100, 500 points, success, and failure). Then, the following contrasts were computed: (a) 100 - 0 points, (b) 500 - 0 points, (c) 500 - 100 points, to identify brain regions activating stronger with point increases (i.e., effect of point magnitude). These were used for the subsequent second-level random-effects model analysis. Second-level statistical parametric maps were produced using a one-sample *t*-test for each group, and group difference analysis was conducted by a two-sample *t*-test. For this analysis, age was included as a regressor of no interest. To identify brain regions recruiting stronger activity with point increases (i.e., effect of point magnitude), we convolved the three point conditions and contrasted them. Finally, small volume correction was conducted for statistical parametric maps based on the striatal, insular, and midbrain regions of interest supplied by WFU Pickatlas version 2.4 (Maldjian et al., 2003).

### Statistical analysis

Statistical analyses of clinical data and correlation analyses between extracted parameter estimates of brain activation and clinical measures were performed using SPSS (SPSS 21.0 for Windows, SPSS Inc., Chicago, IL, USA). For between-group comparison of the mean, distribution normality was confirmed using Kolmogorov–Smirnov tests. Then, normally distributed data and non-normally distributed data were analyzed using *t*-tests and Mann–Whitney's *U*-tests, respectively. For between-group comparison of ratio, χ ^2^-tests were applied. Correlations between activation resulting from one-sample *t*-test in the striatum, insula, and midbrain during reward anticipation (small volume corrected, FWE *p* < 0.05) and duration of illness or abstinence in PG patients were determined by Pearson correlation coefficients. Significance was set at *p* < 0.05 (two-tailed).

## Results

### Demographic data, clinical measures and behavioral performance

Two-sample *t*-tests showed that the two groups did not differ with respect to age (PG: mean = 32.6 years, *SD* = 6.9 years; HC: mean = 33.4 years, *SD* = 8.0 years; *t* = −0.4, *p* = 0.69). χ^2^-tests revealed that there were no between-group differences with respect to handedness (PG: 22 right-handed and 1 left-handed; HC: 26 right-handed and 1 left-handed; *p* = 0.91) and higher education rate (PG: 22 completed high school and 1 did not complete; HC: 27 completed high school and 0 did not complete; *p* = 0.27). Mann–Whitney's *U*-tests showed that the two groups did not differ in terms of task performance (MIDT success rate (%); PG: median = 64.4, range = 45.6–66.7; HC: median = 65.6, range = 46.7–66.7; *p* = 0.08), but PG patients scored higher in gambling severity (SOGS scores: PG: median = 14, range = 10–15; HC: median = 0, range = 0–4; *p* = 0.00), and smoking frequency (FTND; PG: median = 4, range = 0–8; HC: median = 0, range = 0–5; *p* = 0.00) than HC subjects. In addition, PG patients met 6 or more criteria (median 9, range 6–10) in SCI-PG (Grant et al., 2004).

### Imaging results

Because no effect of point magnitude was evident in either group and the 0 point condition also induced activation in reward-related regions, we collapsed the three point conditions (0, 100, and 500 points) into a single regressor.

#### Brain activation during reward anticipation in PG and HC

**Figure 1:**
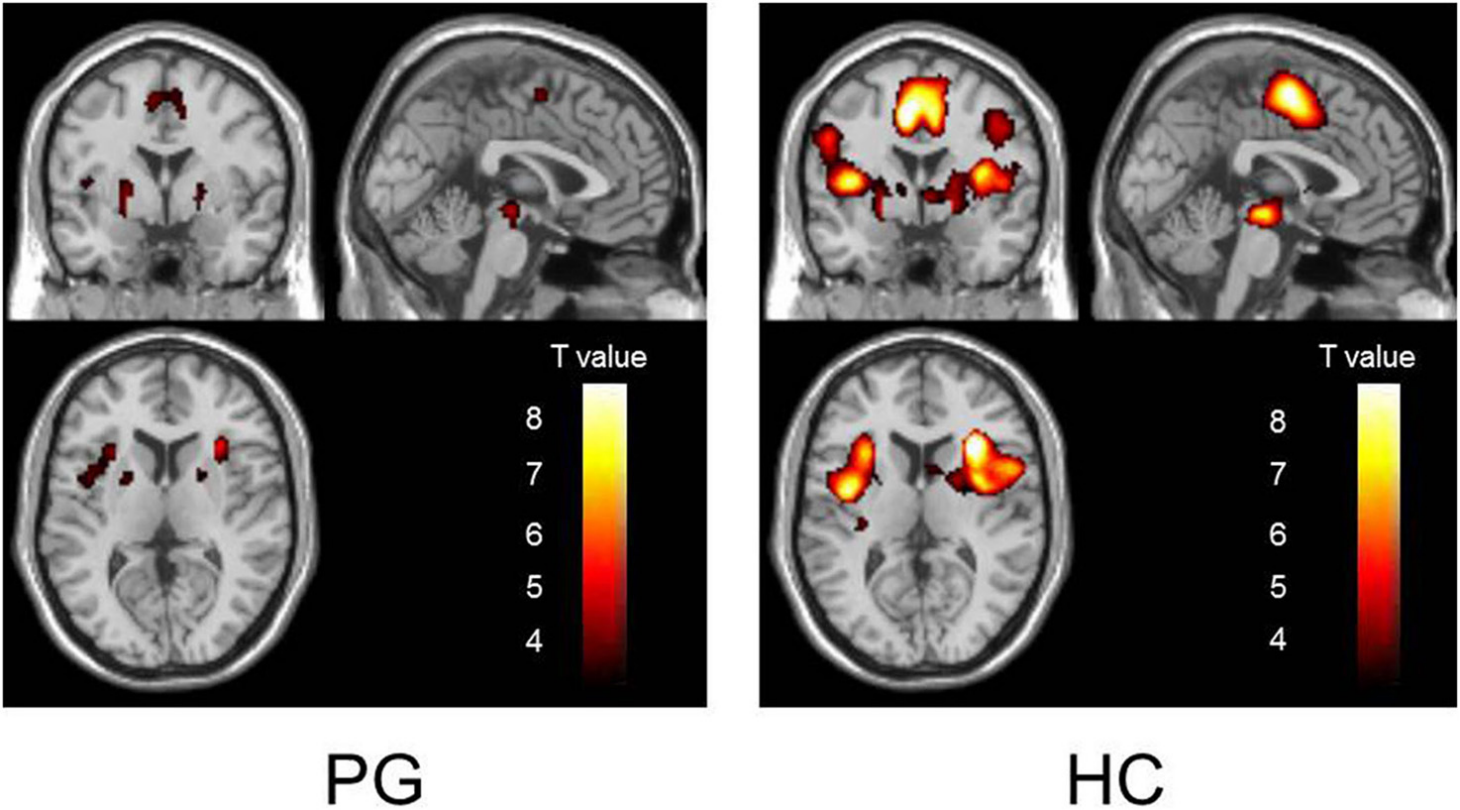
**Activation patterns of each subject group during reward anticipation**. The activation patterns of PG and HC groups during reward anticipation are shown in the left and right panels, respectively (*p* < 0.001, uncorrected). PG patients showed reduced activation compared to HC subjects. Abbreviations: PG, pathological gambling; HC, healthy control.

During reward anticipation, PG patients showed activation in the ventral striatum, cingulate cortex, insula, and brain stem (uncorrected, *p* < 0.001). HC subjects showed spatially similar, but more intense and widespread activations (uncorrected, *p* < 0.001) (Figure 1, Supplementary Table 1) (Coordinates of activated clusters during outcome notification are shown in Supplementary Table 3).

#### Between-group contrast

**Figure 2:**
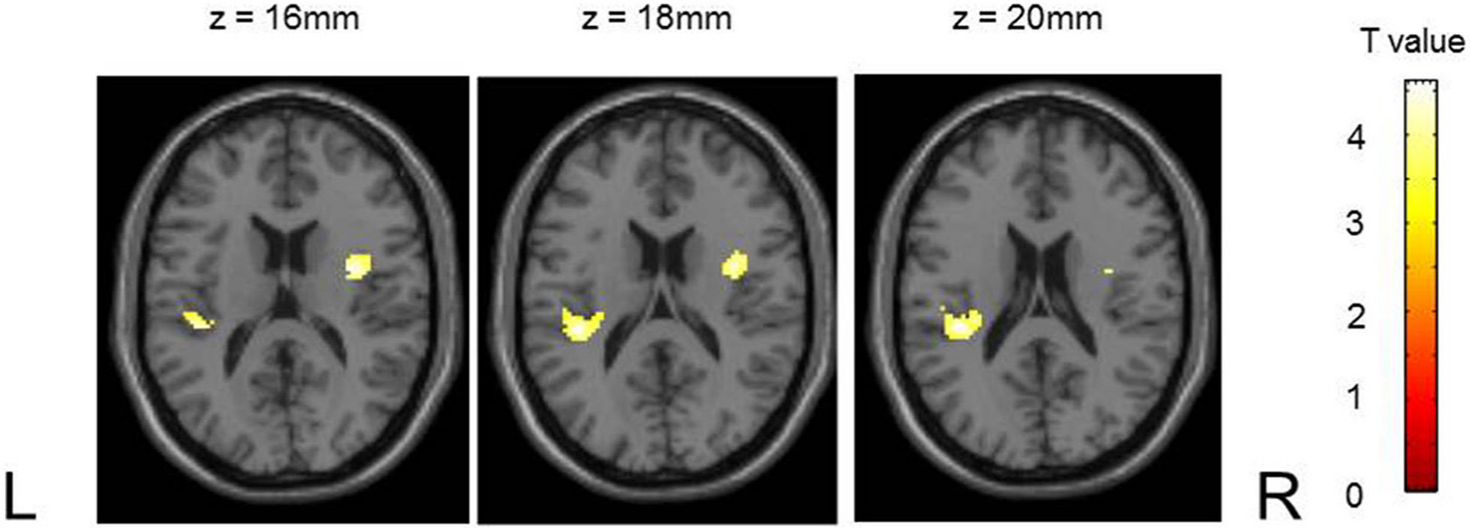
**Differences in activation during reward anticipation in the insula between groups**. Pathological gambling patients showed blunted activation compared to healthy controls (masked by insular region of interest; *p* < 0.05, small volume corrected).

There were no group differences in striatal and midbrain activation during reward anticipation (small volume corrected in the striatum and midbrain, respectively, FWE *p* < 0.05). However, PG patients showed reduced activation in bilateral insula compared to HC subjects (left insula, peak: x, y, z: −38, −32, 18, *k* = 124; right insula, peak: x, y, z: 38, −2, 14, *k* = 112; small volume corrected in insula, FWE *p* < 0.05; Figure 2).

### Correlation analysis

**Figure 3:**
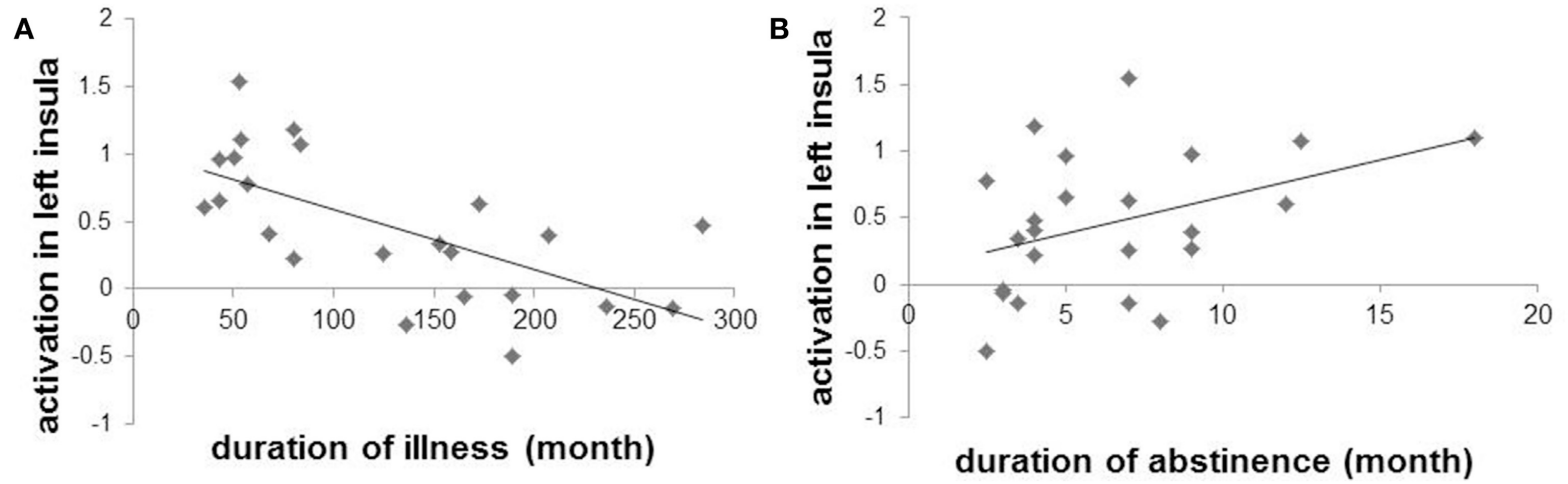
**Scatter plots and regression slopes of activation in left insula and duration of illness (A) or abstinence (B) in pathological gambling patients**.

In PG patients, no significant correlations between duration of illness or abstinence and activation in the bilateral striatum, midbrain or right insula identified in the one-sample *t*-test of PG. However, there was a significant negative correlation between activation in the left insula (peak: x, y, z: −30, 18, 6, *k* = 114; small volume corrected in insula, FWE *p* < 0.05) and duration of illness (*r* = −0.654, *p* = 0.001). Activation in this region also showed a modest positive correlation with duration of abstinence (*r* = 0.401, *p* = 0.058) (Figure 3). There was no significant correlation between duration of illness and duration of abstinence (*r* = −0.297, *p* = 0.169). Some may argue that smoking status may affect insular activation in PG patients. However, the FTND score did not correlate with left insular activation (*r* = 0.306, *p* = 0.155) (Clusters used for correlation analysis are shown in Supplementary Table 2).

## Discussion

PG patients showed blunted activation in the insular cortex during non-specific reward anticipation, as we expected, whereas no group difference was found in the striatum or midbrain. Furthermore, left insular activation in PG patients showed a significant negative correlation with duration of illness, and showed a marginally significant positive correlation with duration of abstinence.

### Activation in striatum

We did not detect between-group differences in striatal activation during non-specific reward anticipation. In previous studies of reward anticipation in PG, patients showed greater striatal activation in response to gamble-like stimuli (e.g., realistic playing cards, images of actual money) (van Holst et al., 2012), less or similar activation in response to monetary reward (Balodis et al., 2012; Sescousse et al., 2013), and blunted activation in response to erotic pictures (Sescousse et al., 2013). This discrepancy between PG's sensitivities to reward can be explained by cue specificity (Leyton and Vezina, 2012). In general, addiction-related cues evoke more striatal activation in addicted populations compared to controls, while non-specific cues induce less activation. This notion can be applied to PG, since it has been shown that, although money is an addiction-related cue for PG patients, indirect cue presentation (i.e., simple text format) may not work as an addiction-related cue (Limbrick-Oldfield et al., 2013). Because we chose “points” as reward, our results were similar to those of indirectly presented monetary reward.

### Activation in insula

In our study, the insula showed activation during reward anticipation in both PG and HC subjects. Although the insula has been mainly reported to be involved with monetary loss, it is also activated during gain anticipation (Samanez-Larkin et al., 2007; Liu et al., 2011). Our findings of insular activation were in line with these previous studies.

Insular activation in response to both positive and negative stimuli is not limited to the context of reward. For example, the insula is activated not only by negative stimuli such as feeling thermal pain (Brooks et al., 2002), seeing a disgusting face or smelling a disgusting odor (Wicker et al., 2003), but also by positive stimuli such as appreciating pleasant music (Koelsch et al., 2006) or experiencing a pleasant taste (O'Doherty et al., 2001). Taken together, these studies support the notion that insular activation is associated with emotional and interoceptive arousal (Critchley et al., 2004; Terasawa et al., 2013). We found that the PG patients showed blunted insular activation during reward anticipation compared to the HC subjects, suggesting that PG patients may have trouble feeling emotional and interoceptive arousal. Investigating subdivisions of insula may also be noreworthy. Activation in the left insula of PG patients extended from anterior insula to mid-insula. Based on recent research investigating insula localization (Deen et al., 2011), activity within the current study appears to be located in the dorsal anterior to middle insula. Dorsal anterior insula and middle insula have tight connections with homeostatic and autonomic control systems, such as hypothalamus, amygdala, dorsal anterior cingulate and ventral striatum (Craig, 2010; Deen et al., 2011). This division is suggested to integrate homeostatic and interoceptive signals (Craig, 2010). Blunted insular activation in PG patients, together with comparable striatal activation, may indicate less emotional and interoceptive arousal in response to equivalently rewarding stimuli, leading PG patients to seek greater reward.

This may provide another point of view for understanding the reward deficiency hypothesis (Blum et al., 1996) beyond the functional impairment of dopamine in the striatum.

Other supporting evidence for a link between low arousal and gambling behavior is that attention deficit/hyperactivity disorder (ADHD) patients, for which treatment arousal-causing medications (e.g., methylphenidate, atomoxetine) are helpful (Corman et al., 2004), often show gambling behavior (Faregh and Derevensky, 2011). In addition, risk indifference by individuals with higher norepinephrine transporters (Takahashi et al., 2013), along with noradrenalic modulation of arousal (Berridge, 2008), suggests that low arousal would underlie risk indifference, leading to gambling behavior. On the other hand, the insula represents risk-related signals such as risk prediction and risk prediction error (Preuschoff et al., 2008). Risk seekers show less activation in the insula during a high-risk gamble task compared to risk averters (Rudorf et al., 2012). Taken together, decreased insular activity during reward anticipation in risk-taking PG patients (Ligneul et al., 2013) may indicate a neural representation of indifference to risk.

### Relationship between insular activation and clinical characteristics of PG

In PG patients, activation in the left insula decreased with increasing duration of illness and increased marginally with increasing duration of abstinence. These effects suggest that the relatively decreased activity in the insula is resulting from excessive gambling behavior, and might be recovered by abstinence. Detailed mechanisms of behavioral addiction are less commonly investigated. One possible reason for this is the difficulty of establishing animal models of behavioral addiction. To our best knowledge, there is only one animal study on behavioral addiction, which showed that chronic exposure to a gambling-like schedule of reward predictive stimuli can promote sensitization to amphetamine in rats (Zack et al., 2014). Similarly, a recent human PET study reported greater amphetamine-induced dopamine release in PG patients (Boileau et al., 2013). Thus, precise and detailed molecular mechanisms of behavioral addiction are not fully understood. However, based on the above-mentioned circumstantial evidence, it is reasonable to assume that within dopamine-mediated circuits, cellar and molecular adaptation are commonly occurrences in behavioral addiction (see Nestler, 2005 for the common mechanisms). Because the insula receives moderate dopaminergic innervation (Ito et al., 2008), dopamine-mediated adaptation might in part underlie the altered insula activation in PG patients.

Decreased insular activity has been related to a relapse in methamphetamine-dependent patients (Paulus et al., 2005), and in alcohol-dependent patients (Seo et al., 2013). According to the results of these studies of substance use disorders, along with our findings from non-substance addiction, activity in the insula might serve as an important clinical marker of addiction itself.

### Limitations and strengths

There are several limitations to our study. First, although there are many important structures involved with addiction, we could detect activation difference only in the insula. The lack of group difference in reward-related regions might stem from the task we used. For ethical reasons, we decided not to use real money and/or gamble-related stimuli in abstinent and treatment-seeking patients. Concerns that such provocative stimuli might trigger craving or relapse were expressed by the treatment facility as well as the ethics committee. A previous study of gamblers (mainly recreational gamblers) reported that midbrain activation during near-miss with slot machines predicted gambling severity (Chase and Clark, 2010). Careful interpretation is needed due to the fact that the type of stimuli and clinical stage (recreational, pathological, and recovered) of patients will influence the reward-related region's activation. The fact that we did not use real money in the MIDT might have contributed to an additional limitation for why the magnitude effect of the point condition, including the 0 point condition, was not observed. Second, smoking was more prevalent and comorbid disorders were found among PG patients. Thus, the effects of smoking and comorbid disorders on brain activation could not be ruled out. However, a high smoking rate and comorbid disorders are common in PG, which may support that our PG sample represents a general PG sample. Third, this study was conducted using a cross-sectional design. A longitudinal study will be needed to investigate the effects of abstinence on brain activity more directly. Fourth, the possibility that repeated risk computation during frequent gambling behavior might affect insular activity cannot be ruled out. Future studies should include recreational gamblers (i.e., non-addicted gamblers) as well. Finally, although “Pachinko” (a kind of gaming machine) is the most popular form of gambling in Japan, the machine is not common in any countries other than Japan. Thus, it may be difficult to generalize from our results to studies of PG in other countries. Notwithstanding these limitations, a strength of our study is the homogeneity of our PG patients. The number of gaming machines is remarkably high in Japan (http://www.gamingta.com/pdf/World_Count_2014.pdf), and all our PG patients engaged in machine gambling. Moreover, because illicit drug availability is strictly controlled, the prevalence of illicit drug abuse is much lower in Japan (Yamamoto, 2004). Accordingly, the possibility that illicit drugs might affect neural activation is very low. In addition, all PG patients were recruited from a treatment facility where use of illicit drugs would be almost impossible. Furthermore, although PG patients in previous studies were ongoing gamblers, all PG patients in this study were abstinent from gambling, allowing us to exclude the effects of ongoing gambling behaviors on brain activity.

## Conclusion

We found that altered insular activation during reward anticipation in PG patients reflects the duration of illness and abstinence. Studies on behavioral addictions including PG could contribute to disentangling the complex features of addiction by parsing the direct toxicity of addictive substances. Thus, altered insular activation may serve as a clinical marker of addictive behavior *per se*, and may be a potential target for a better understanding of the pathophysiology of addiction.

### Conflict of interest statement

The authors declare that the research was conducted in the absence of any commercial or financial relationships that could be construed as a potential conflict of interest.
