# Supplementary material for: Insular activation during reward anticipation reflects duration of illness in abstinent pathological gamblers
Source: Front Psychol. 2014 Sep 9;5:1013. doi: 10.3389/fpsyg.2014.01013 (PMC4158979; doi:10.3389/fpsyg.2014.01013)
Supplement: Supplementary file 1 [file DataSheet1.DOCX]

***Supplementary Material***

**Insular activation during reward anticipation reflects duration of illness in abstinent pathological gamblers**

**Kosuke Tsurumi^1^, Ryosaku Kawada^1^, Naoto Yokoyama^1^, Genichi Sugihara^1^, Nobukatsu Sawamoto^2^, Toshihiko Aso^3^, Hidenao Fukuyama^3^, Toshiya Murai^1^, Hidehiko Takahashi^1^***

^1^Department of Psychiatry, Kyoto University Graduate School of Medicine, Kyoto, Japan

^2^Department of Neurology, Kyoto University Graduate School of Medicine, Kyoto, Japan

^3^Human Brain Research Center, Kyoto University Graduate School of Medicine, Kyoto, Japan

*** Correspondence:** Hidehiko Takahashi, Department of Psychiatry, Kyoto University Graduate School of Medicine, 54 Shogoin-Kawahara-cho, Sakyo-ku, Kyoto, 6068507, Japan.

hidehiko@kuhp.kyoto-u.ac.jp

1. **Supplementary Data**
   1. Additional analysis of between-groups contrast

In order to examine activation differences between groups in the brainstem and amygdala, we conducted small volume correction in both regions. We did not detect any activation differences in the brainstem or amygdala during reward anticipation (small volume-corrected in brainstem and amygdala, respectively, FWE p < 0.05).

- 1. Activations during outcome notification

We computed the following two more contrasts: (a) success minus failure, (b) failure minus success, to identify brain regions activating more strongly due to different outcome notifications. These were then used for the subsequent second-level random-effects model analysis. Second-level statistical parametric maps were produced using a two-sample t-test between the PG group and HC group. For this analysis, age was included as a regressor of no interest. To identify brain regions activating more strongly due to different outcome notifications, we convolved the two outcome conditions and contrasted them. Success notification versus failure notification activated the bilateral anterior cingulate cortex, posterior cingulate cortex, frontal and parietal regions in PG patients, and the bilateral anterior cingulate cortex, frontal and parietal regions in HC subjects (Table S2a). On the other hand, failure notification versus success notification activated right frontal regions in PG patients, and bilateral frontal regions in HC subjects (Table S2b). There were no group differences in the two conditions.

1. **Supplementary Tables**

**Supplementary Table 1. Brain regions of the two groups activated during reward anticipation**

Coordinates of clusters corresponding to Figure 1 are shown (k > 100, p < 0.001, uncorrected). Abbreviations: PG, pathological gambling; HC, healthy control; rt, right; lt, left; bil, bilateral; MNI, Montreal Neurological Institute.

|  |  |  | **MNI coordinates** | | |  |  |  |  |
| --- | --- | --- | --- | --- | --- | --- | --- | --- | --- |
|  | **Structure** |  | **x** | **y** | **z** |  | **k** |  | **T/Z value** |
| **PG** | rt. putamen |  | 18 | 6 | -2 |  | 205 |  | 5.54/4.83 |
|  | rt. insula |  | 32 | 20 | 8 |  | 115 |  | 5.36/4.71 |
|  | lt, putamen |  | -20 | 6 | -4 |  | 237 |  | 5.10/4.53 |
|  | lt. precentral gyrus |  | -32 | -24 | 52 |  | 200 |  | 4.86/4.36 |
|  | bil. thalamus/ bil. midbrain |  | 2 | -20 | -10 |  | 344 |  | 4.83/4.33 |
|  | bil. cingulate cortex |  | -4 | -6 | 58 |  | 404 |  | 4.77/4.29 |
|  | lt. insula |  | -30 | 18 | 6 |  | 174 |  | 4.26/3.90 |
|  |  |  |  |  |  |  |  |  |  |
| **HC** | lt. insula/ bil. striatum/  bil. midbrain |  | 32 | 22 | 8 |  | 5288 |  | T=12.80 |
|  | bil. cingulate gyrus |  | -6 | -4 | 48 |  | 7232 |  | 11.32/7.81 |
|  | rt. insula |  | -42 | -4 | 12 |  | 2451 |  | 8.53/6.60 |
|  | rt. inferior parietal cortex |  | 56 | -18 | 24 |  | 242 |  | 6.25/5.30 |

**Supplementary Table 2. Brain regions of the two groups activated during outcome notification**

Coordinates of activated clusters of each group during outcome notification are shown (k > 100, p < 0.001, uncorrected); (a) shows activation patterns of success notification versus failure notification and (b) shows those of failure notification versus success notification.

1. Success notification versus failure notification

|  |  |  |  | **MNI coordinates** | | |  |  |  |  |
| --- | --- | --- | --- | --- | --- | --- | --- | --- | --- | --- |
|  |  | **Structure** |  | **x** | **y** | **z** |  | **k** |  | **T/Z value** |
| **PG** |  | bil. anterior cingulate gyrus |  | -10 | 34 | -12 |  | 3273 |  | 6.99/5.76 |
|  |  | lt. superior frontal gyrus |  | -18 | 42 | 46 |  | 1850 |  | 6.33/5.35 |
|  |  | lt. inferior parietal cortex |  | -38 | -68 | 34 |  | 801 |  | 5.04/4.49 |
|  |  | bil. posterior cingulate cortex |  | -4 | -50 | 24 |  | 1728 |  | 4.97/4.43 |
|  |  | rt. superior temporal gyrus |  | 50 | -62 | 28 |  | 119 |  | 4.19/3.84 |
|  |  |  |  |  |  |  |  |  |  |  |
| **HC** |  | bil. anterior cingulate gyrus |  | -8 | 36 | -12 |  | 1170 |  | 6.74/5.61 |
|  |  | lt. middle frontal gyrus |  | -24 | 20 | 58 |  | 1040 |  | 5.53/4.83 |
|  |  | lt. paracentral lobule |  | -14 | -28 | 54 |  | 131 |  | 4.66/4.21 |
|  |  | lt. inferior parietal cortex |  | -42 | -62 | 44 |  | 127 |  | 4.38/3.99 |
|  |  | rt. middle frontal gyrus |  | 12 | 38 | 24 |  | 103 |  | 4.19/3.84 |
|  |  | rt. superior frontal gyrus |  | 18 | 42 | 50 |  | 182 |  | 4.05/3.73 |

1. Failure notification versus success notification

|  |  |  |  | **MNI coordinates** | | |  | |  | |  | |  | |  |
| --- | --- | --- | --- | --- | --- | --- | --- | --- | --- | --- | --- | --- | --- | --- | --- |
|  |  | **Structure** |  | **x** | **y** | **z** | |  | | **k** | |  | | **T/Z value** | |
| **PG** |  | rt. inferior frontal gyrus |  | 42 | 20 | -6 | |  | | 137 | |  | | 4.22/3.87 | |
|  |  |  |  |  |  |  | |  | |  | |  | |  | |
| **HC** |  | rt. superior frontal gyrus |  | 6 | 14 | 66 | |  | | 154 | |  | | 4.54/4.11 | |
|  |  | rt. inferior frontal gyrus |  | 44 | 22 | 2 | |  | | 118 | |  | | 4.04/3.72 | |

**Supplementary Table 3. Brain regions of PG patients remained significant after small volume correction in striatum, insula, and midbrain.** Clusters used for correlation analysis are shown (small volume-corrected in striatum or insula, FWE p < 0.05).

|  |  | **MNI coordinates** | | |  |  |  |  |
| --- | --- | --- | --- | --- | --- | --- | --- | --- |
| **Structure** |  | **x** | **y** | **z** |  | **k** |  | **T/Z value** |
| rt. striatum |  | 18 | 6 | -2 |  | 179 |  | 5.54/4.83 |
| lt. striatum |  | -20 | 6 | -4 |  | 226 |  | 5.10/4.53 |
| rt. insula |  | 32 | 20 | 8 |  | 72 |  | 5.36/4.71 |
| lt. insula |  | -30 | 18 | 6 |  | 114 |  | 4.26/3.90 |
| bil. midbrain |  | 2 | -20 | -10 |  | 318 |  | 4.83/4.33 |
